# Thermal Stability of Amorphous Solid Dispersions

**DOI:** 10.3390/molecules26010238

**Published:** 2021-01-05

**Authors:** Dijana Jelić

**Affiliations:** Chemistry Department, Faculty of Natural Sciences and Mathematics, University of Banja Luka, dr Mladena Stojanovića 2a, 78 000 Banja Luka, Bosnia and Herzegovina; dijana.jelic@med.unibl.org

**Keywords:** amorphous solid dispersion of drug, thermal stability, polymers, kinetics, kinetic analysis

## Abstract

Amorphous solid dispersion drug delivery systems (ASD DDS) were proved to be efficient for the enhancement of solubility and bioavailability of poorly water-soluble drugs. One of the major keys for successful preparation of ASD is the selection of appropriate excipients, mostly polymers, which have a crucial role in improving drug solubility and its physical stability. Even though, excipients should be chemically inert, there is some evidence that polymers can affect the thermal stability of active pharmaceutical ingredients (API). The thermal stability of a drug is closely related to the shelf-life of pharmaceutical products and therefore it is a matter of high pharmaceutical relevance. An overview of thermal stability of amorphous solids is provided in this paper. Evaluation of thermal stability of amorphous solid dispersion is perceived from the physicochemical perspective, from a kinetic (motions) and thermodynamic (energy) point of view, focusing on activation energy and fragility, as well all other relevant parameters for ASD design, with a glance on computational kinetic analysis of solid-state decomposition.

## 1. Introduction

### 1.1. What Is the Thermal Stability of Drugs? 

Thermal stability is a characteristic of a material to retain its structure and properties when exposed to higher temperatures. The stability of pharmaceutical compounds, in general, can be physical, chemical, and biological. The thermal stability of drugs mutually correlates with each other. Physical instability is mostly related to the mobility of molecules, due to temperature increment, while chemical instability is related to energy (heat) flow which can induce thermal decomposition of drugs. These changes might lead to a decreased pharmacological activity and shortened shelf life of drugs. The World Health Organization (WHO) recommends that the chemical and thermal stability of products should be evaluated to identify any degradation species in final medicinal products [1,2]. Hence, understanding the response of drugs, excipients and their formulations to thermal stresses is undoubtedly an inseparable part of the development of drug products [3]. Evidently, the thermal stability of drugs is a matter of high pharmaceutical relevance. 

The thermal stability of amorphous pharmaceutical compounds is the focus of this paper. The amorphous state (AS), of a drug is an intriguing one since it improves the solubility of a poorly water-soluble drugs [4]. “An estimated 40% of approved drugs and nearly 90% of the developmental pipeline drugs consist of poorly soluble molecules. Several marketed drugs suffer from poor solubility” [5].

The amorphous state along with crystal state (CS) belongs to the solid-state. Crystal state has a long-range order molecule packing, with a characteristic melting point (*T_m_*), and excellent stability, but from a pharmaceutical point of view, suffers from low solubility and therefore low bioavailability. On the other hand, amorphous state is characterized by a good amount of energy that provides good drug solubility and enhanced bioavailability. A disadvantage of possessing such a high amount of free energy is the instability of the amorphous state [4]. As a consequence, the recrystallization process occurs. Nucleation and crystal growth are parts of the crystallization process [6]. Amorphous solid dispersions, in which some amorphous drug is dispersed in the polymeric matrix is an effective approach to increase the stability of amorphous form through inhibition of crystallization. Transition from the AS state (unstable) towards the CS state (stable) stands in correlation with thermodynamics, kinetics, and drug-excipient interactions. Knowledge of thermal behavior, especially the kinetics, leads to lifetime prediction [7,8], which is essential for prediction of the storage conditions of drugs, as well as for technological processing. 

What can we learn from thermal stability data? First, the onset temperature of degradation that goes in favor of the thermal behavior of drug can be determined [3]; Second, we can get insight into the mechanism of thermal decomposition of drugs and potential degradants (i.e., the relationship between the molecular structure and thermal stability) [7]; Third, understanding of compatibility/incompatibility between drug and excipients is revealed [8]; Fourth, we learn about its solubility potential [9]; Finally, information on handling/storage of drugs, shelf-life, and usage can be provided [10].

There are excellent reviews and research papers published so far [9,11,12,13,14,15,16,17,18], regarding the stability of amorphous drugs as well as insoluble drug delivery strategies. Selection of proper and suitable polymers, their miscibility, and interactions with a drug are favored approaches. The hypothesis that the polymer carrier can affect the thermal stability of drugs in ASD was proven recently and, one should not take for granted that the drug-polymer interaction indeed affects the thermal stability of the drug [19]. Keeping in mind that thermal instability of drugs can lead to a diminished pharmacological activity, a shortened shelf-life, and a potential harmful product, thermal stability of drugs deserves a to be reviewed and further explained. Hence, this paper provides an overview of the quantitative meaning of thermal stability considering kinetics and thermodynamics factors relevant to predict and control stability and, the shelf life of amorphous solid dispersions.

### 1.2. Kinetic Analysis of Solid State

In order to quantify thermal stability, knowledge of Arrhenius parameters (constant rate (*k*), activation energy (Ea), preexponential factor (*A*)) and a suitable mechanism *f*(*α*) are of great interest. Computational kinetic analysis (CKA) of the solid-state decomposition process with a mission to determine the parameters of the Arrhenius equation (*k* = *Ae* − *E*/*RT*) and the mechanism of thermal decomposition is an emerging topic [20,21,22,23,24]. The practical aspect of kinetic analysis lies in the prediction of material (drug) lifetime. Considering that thermal decomposition of solids is quite challenging, a significant amount of literature is available from ICTAC (International Confederation of Thermal Analysis and Calorimetry) kinetics and lifetime project (2000) about computational aspects of solid-state kinetic analysis, presenting both benefits and shortcomings (or even criticism) of solid-state kinetic analysis [25,26].

The purpose of kinetic analysis is to establish a mathematical relationship between constant rate (*k*), conversion degree (*α*), and the temperature (*T*), in other words, to determine the so-called kinetic triplet (*A*, Ea, and *f*(*α*)) for each single step. A common starting point in the computational kinetic analysis based on thermal analysis data is the following equation [25,26,27,28]:

(1) $$\beta \frac{d\alpha }{dt}=kf\left(\alpha \right)=Ae^{-E/RT}f(\alpha )$$

where *α* is the conversion degree, *t* is the time, *k* is the constant rate from Arrhenius expression

(2) $$k=Ae^{-\frac{E_{}}{RT}}$$

*E* is the apparent activation energy, *A* is the preexponential factor, *R* is the gas constant, *f*(*α*) is the mathematical expression dependent on the reaction mechanism and *β* is the heating rate.

The mass loss of a solid reactant is always expressed in terms of conversion degree, *α* or often called, extent of conversion, which ranges from 0 to 1 and is expressed by the following equation:

(3) $$\alpha =\frac{m_{o}-m_{t}}{m_{o}-m_{\infty }}$$

where *m_o_* is the initial mass of the sample, *m_t_* is the sample mass at some time *t* and *m*_∞_ is the final mass of the sample.

The integration of Equation (1) leads to Equation (4) in which *g*(*α*) is an integral form of the reaction model.

(4) $$g(\alpha )=\frac{A}{\beta }{\int_{0}^{T}e}^{-\frac{E}{RT}}dT$$

Kinetic analysis can be performed in isothermal (*T* = const) and non-isothermal conditions (*T* = *T*(*t*)). Both approaches are considered accurate, even though isothermal runs have a limited temperature range [29]. In 2011 ICTAC gave recommendations that at least three different heating rates are acquired for the investigation of activation energy [29]. There are two approaches for kinetic analysis, isoconversional and model-fitting methods. Isoconversional methods (e.g., Friedman, Coast-Redfern, Kissinger, Flynn-Wall-Ozawa) evaluate the activation energy without assumptions about the reaction model and they are often called the model-free method [29,30]. Even though, these methods do not identify the reaction model, some predictions and clues about the reaction model should be assumed. The model fitting approach provides us with a reaction model that fits the best decomposition process, such as nucleation and crystal growth, diffusional, contracting geometry and reaction-order models (Table 1). The kinetic parameters associated with a specific reaction assume to represent the conversion dependence of the reaction rate [31]. It is difficult to determine whether the isoconversional or model-fitting method would provide more accurate predictions, therefore by using both the greatest reliability is achieved [32]. It should be noted that thermal decomposition of solid is quite a complex process [33,34] and determination of the kinetic parameters for the thermal decomposition is not always straightforward. For a successful kinetic analysis, examination of different kinetic calculation methods should go along with rigorous physicochemical insight on the process. Nowadays, specific commercial software programmes for calculating kinetics parameters such as Kinetics Neo (Netzsch), AKTS (Advanced Kinetic and Technology Solution), Kinetics2015 (GeoIsoChem) are available and can assist researchers in the field of kinetic analysis of the solid state. Since the scope of this review is on the thermal stability of drugs, readers interest in further details about solid-state kinetics can refer to the following references [25,26,28,29,30,31,32,33,34,35,36,37,38]

Activation energy, the most used Arrhenius parameter, is often applied to justify the thermal stability of drugs. Activation energy is very important for a fruitful reaction and it must be measured for the evaluation of thermal stability. If Ea keeps a constant value over the entire α range and if there are no curves on the reaction rate curve, one can assume it is a single step process. If Ea varies a lot with α, and the constant rate graph have peaks, it is a multi-step reaction [26,36]. Generally, the greater the activation energy, the greater is the thermal stability.

Wassel et al. kinetically investigated the thermal stability of three anti-inflammatory drugs: diflunisal, tenoxicam, and celecoxib. Based on the kinetic parameters value which showed a significant difference, the thermal stability of drugs was estimated in the following order: diflunisal > tenoxicam > celecoxib (220.7 kJ/mol, 151.4 kJ/mol, and 101.22 kJ/mol, respectively) [39]. For two antihypertensive drugs valsartan and losartan potassium, Ea was quantified to be 50 kJ/mol and 250 kJ/mol, respectively, indicating a greater thermal stability for a drug with a higher value of Ea [40]. However, it should be noted that thermal instability did not only occur at the expanse of an Ea decrease but also sometimes at the expanse of increasing preexponential factor A. Osman et al. reported that thermal instability of nifedipine (NIF) with PVP in amorphous solid dispersion was associated with an increas in the preexponential factor due to a rise in the entropy of activation. For the quantification of the thermal stability of NIF-PVP ASD, a constant rate ratio of solid dispersion and neat drug at a given temperature was also used [19]. Jelic et al. explored the stability of indomethacin solid dispersions having different ration of PVP (1:1, 1:7, 1:10, and 1:20). It was concluded that depending on the amount of PVP in solid dispersion thermal stability is guided differently and, for the first two systems (1:1 and 1:7) thermal stability of indomethacin is accomplished by an increase in activation energy, while for the 1:10 and 1:20 system, thermal destabilization was connected with a decrease in the pre-exponential factor [41].

Therefore, only considering the Ea for thermal stability evaluation can be misleading. Overall, in order to properly evaluate the thermal stability of drugs, it is important to have a meaningful interpretation of kinetic results that relies on physicochemical indications. It should also be considered that some processes have a non-Arrhenius temperature dependence, that might arise due to a change in the dominant mechanism [42]. For example, the Arrhenius equation can give information for the chemical degradation of a drug at storage temperature by extrapolating data obtained at higher temperatures, but in the case of the crystallization process, which is more physical in nature, this approach is questionable since onset formation of crystals do not follow the Arrhenius temperature dependence. Furthermore, Arrhenius linearity is questionable in case of complex reaction mechanism, uncontrolled humidity, and other phase transitions such as vaporization or melting [43]. Ideally, Arrhenius dependence should only be determined within an “isokinetic” range for a given compound [44,45,46].

Thermal stability of different classes of drugs such as anti-cancer [47], vitamin [48], antimalarial [49], antihypertensive [50], antibiotic [51], and anti-HIV drugs [52] was widely evaluated in the literature by the proposed CKA approach using thermal analysis techniques (Table 2) The studies on kinetics, as well as mechanism of thermal decomposition of drugs have important theoretical significance to obtain information on the thermal stability of drugs, and further understanding of the relationship between thermal stability and molecular structure.

The thermal stability of drugs could also be altered by the influence of the excipients on the decomposition mechanism of the pure drug API [60]. Changes in the thermal properties of drugs, such as onset temperature, melting point, or some thermal event (exothermic/endothermic) are detectable signs for the disturbed thermal stability of drugs. Seiman et al. evaluated the thermal stability of an anti-hypertensive drug, amlodipine besylate (AB), in its pure state and in binary mixture with excipients. Amlodipine besylate has a thermal sensitive structure and it melts in the temperature region 175–220 °C. Thermal studies showed that thermal decomposition of amlodipine besylate is a complex one and is developing through two dehydration processes. The first dehydration process happens due to loss of water of crystallization (117.5 kJ/mol) and the second process owns to the dehydration process of maleic acid (210 kJ/mol). Since the presence of excipients can affect thermal stability, potential interactions between amlodipine besylate and the excipients were investigated in the 175–220 °C and no such interaction was observed. Hence, neither the decomposition process or the interaction with the excipients affects the thermal stability of amlodipine besylate [61]. A similar investigation was carried out by Tomasseti et al., in which the thermal stability of acetominophen was investigated [62]. It was a one-step decomposition process and unlike the previous case, Tomasseti et al. showed that the thermal stability of acetominophen was greatly disturbed, due to the incompatibility between acetominophen and mannitol, which proved an important role of excipients in drug formulation. Serajjudin et al. also reported the degradation of an antihypertensive drug into three degradation products induced by the presence of excipient magnesium stearate [63]. There are also some reports on the change of thermal stability of excipients by other excipients [64,65]. Leite et al. conducted a valuable study for the prediction of bioavailability problems of poorly water soluble drugs by estimating the thermal stability of nifedipine crystals in different solvents. Thermal stability of nifedipine was evaluated as raw material and in different solvents such as methanol (130.7 kJ/mol, 27.12 min^−1^), isopropyl alcohol (131.5 kJ/mol, 27.02 min^−1^), acetone (126.2 kJ/mol, 26.11 min^−1^), ethyl acetate (124.9 kJ/mol, 25.59 min^−1^), chloroform (130.9 kJ/mol, 26.91 min^−1^), and dichloromethane (127.7 kJ/mol, 26.62 min^−1^). The nifedipine crystals obtained in methanol were thermally the least stable [66]. Buda et al. even proposed the development of a novel generic solid pharmaceutical formulation of perindopril erbumine since increased thermal stability occurred due to the presence of magnesium stearate, lactose, anhydrous colloidal silica, and microcrystalline cellulose [50].

### 1.3. Quantification of Stability as an Important Tool for ASD Design

The pharmaceutical scientific community developed different strategies to overcome the poor solubility of drugs, such as particle size reduction and nanonization; amorphous solid dispersion; polymeric micelles; pH modification and salt formation; co-solvency and surfactant solubilization; solid lipid nanoparticles; liposomes, proliposomes and microemulsion; and self-emulsifying drug delivery systems and inclusion complexation [67,68,69,70,71,72]. Among these strategies, ASD showed commercial success and is a promising concept for improving bioavailability. Great attention is set on the physical stability during ASD design since ASD are susceptible to the recrystallization process during the storage period or even after oral administration. Polymers have multiple effects in ASD formulation: to slow or inhibit crystallization kinetics, to absorb into the drug surface to prevent the nucleation process, and to interact with the drug substance [73]. Choosing a proper and convenient polymer for ASD systems is a significant and challenging task due to lack of data on drug solubility in polymeric matrices and problems associated with high viscosity of polymers. The number of polymers used as carriers in ASD is quite limited. Among the most used carriers are some low molecular weight molecules such as urea, sugars, and polymers including PVP and polyethylene glycol. The most recently used ones are acetate-succinate derivates of HPMC and the latest, dating from 2007 is Solplus [74]. In order to choose an appropriate polymer for ASD both kinetical and thermodynamical approach must be considered. The former is related to mobility and the latter refers to solubility. Hydrophilic polymers such as PVP or HPMC are often used in ASD formulation, but one should keep in mind that these polymers, being more polar than the neat amorphous drug, have an affinity to moisture that can relieve the crystallization process [21]. Since the crystallization process affects the solubility and dissolution rate, the polymers’ role is to inhibit the crystallization process. Aso et al. reported that the presence of 10% of PVP slows the rate of crystallization of nifedipine by a factor of 300 [75]. It is worth mentioning that molecular mobility in ASD have several types, starting from non-Arrhenius relaxation α, that is responsible for the gel-state at the *T_g_* (glass transition temperature), and a set of few secondary relaxations that are inter- or intra-molecular origin named β, γ, δ, etc. reflecting the localized rapid motions [76]. All these processes have a different activation energy and therefore the kinetics of crystal nucleation in a solid-state is complex and is often not well understood. Probably the key issue in understanding the nucleation mechanism is to find what encourages the nucleation and growth phenomena.

Solid-state kinetics presented in the previous chapter might be used as a practical tool for assessment of the recrystallization mechanism which can contribute to a better understanding of the nucleation and crystal growth mechanism of amorphous forms since activation energy (a quantitative measure of thermal stability) is responsible for the development of stable nuclei and molecules diffusion. Therefore, establishing a protocol that will allow quantification of prediction of long-time stability of amorphous forms might lead to robust ASD formulation and could save time, money, and human resources.

Crystallization, an unfavorable exothermic phase transformation in ASD, proceeds through two steps: nucleation (*N*) and crystal growth (*CG*) [77]. These two stages can be expressed by the following equations:

(5) $$N=Ae^{-}{}^{\left(\frac{\Delta G_{D}+\Delta G_{K}}{RT}\right)}$$

(6) $$CG=\frac{k}{\eta }\left[1-e^{-\left(\frac{\Delta G_{D}}{RT}\right)}\right]$$

Both Equations (5) and (6) consist of kinetic and thermodynamic factors. In Equation (5) Δ*G_D_* is the Gibbs free energy, which is a thermodynamic term related to the formation of a nucleus, Δ*G_K_* is Gibbs free energy, a kinetic term, responsible for molecules motions and *R* is the universal gas constant. In Equation (6) *η* is viscosity, while 1/*η* denotes mobility; the expression

(7) $$1-\exp\frac{\Delta G_{v}}{RT}$$

is the thermodynamic factor responsible for crystal growth, *k* is the constant and Δ*G_v_* is the free energy difference between the amorphous and crystalline state. A similar expression was given by Cohen et al., who accounted for the thermodynamic part via the Arrhenius equation [78].

(8) $$k_{T}=Ae^{\left[\frac{Ea}{RT}-\left(\frac{V}{V_{f}}\right)\right]}$$

where *V* is the critical volume for molecular mobility and *V_f_* is the free volume, *Ea* in the mathematical expression presents activation energy, the main barrier for the crystallization process. It should be noted that when *V_f_* >> *V*, the term in the brackets of Equation (8) approaches zero, and Equation (8) becomes the original Arrhenius equation. Cohen et al. used this equation for the evaluation of molecular transport in liquid and glasses. Genton et al. explored the effect of temperature and humidity (Equation (7)) on nitrazepam solid dispersion stability also utilizing the Arrhenius equation but modified with the term that accounts for humidity [79].

(9) $$\ln k_{T,h}=\ln A-\frac{Ea}{RT}+bh$$

where *k* is a constant rate at a specific temperature and relative humidity, *b* is the moisture sensitivity term independent of temperature, *Ea* is an apparent activation energy and *A* is the Arrhenius collision frequency.

Equation (9) was previously used by Watermann et al. for solid-state pharmaceuticals through the concept called isoconversion paradigm for faster prediction for drug substance and drug product shelf-life [43]. Zhu et al. reported that the crystallization process of pure amorphous ritonavir (RTV) and amorphous solid dispersion of ritonavir and hydroxypropylmethylcellulose acetate succinate (RTV-HPMC ASD) can be quantitatively described by Equation (9) based on the *Ea* and *b* parameter. The stability of RTV-HPMC ASD proved to be governed by the nucleation kinetic process [21]. Zhu et al. further modified the Arrhenius equation to account for the induction and crystallization time (Equation (10)) and gave one new and improved kinetic approach that allowed estimation of long-term stability of efavirenz and PVP in a cost- and time-effective manner. The new kinetic model finds its roots in the classical Avrami model (refer to Table 1), which is one of the most used models for the solid-state recrystallization processes [6].

(10) $$\alpha \left(t\right)=1-\exp\left(-kt^{n}\right)$$

where *α*(*t*) is the relative crystallinity, *k* presents the crystallization rate and *n* is the Avrami exponent. The rate constant, *k* is introduced via the Arrhenius equation. The rising problem with Equation (10) is that the nucleation rate, described as the energy difference between the crystalline and amorphous state, has a constant value. This can attribute to some misleading recrystallization rates. In order to improve Equation (10), Yang et al. introduced an improved description of nucleation rate *J*(*t*) which is proportional to the amorphous fraction 1 − *α*(*t*) (Equation (11)) [73] and it is governed by the activation energy, Ea [80].

(11) $$J\left(t\right)=J_{0}\left(1-\alpha \left(t\right)\right)$$

(12) $$J\left(t\right)=J_{0}\left(-\frac{\Delta G^{*}}{RT}\right)=A\exp\left(-\frac{E_{a}}{RT}\right)\exp\left(-\frac{\Delta G^{*}}{RT}\right)$$

where ΔG* is the thermodynamic barrier related to interfacial energy, *E_a_* is the apparent activation energy, *A* is the pre-exponential factor related to ion diffusion and nuclei surface properties and *J*_0_ is the initial nucleation rate. Unfortunately, the determination of *J*_0_ is quite limited, mostly based on theoretical predictions and a few experimental data [81]. The lack of *J*_0_ data is probably due to assumptions that *J*_0_ does not affect the nucleation rate a great deal, even Li et al. and Wallace et al. confirmed that the initial nucleation rate can make a 10 times difference in the nucleation rate under certain circumstances [80,82].

Nucleation as the initial stage of the crystallization process is followed by crystal growth (CG). In the literature, three models are proposed for observation of CG: homogeneous nucleation-based crystallization; tension-induced interfacial mobility and, solid-state crystal growth by local mobility (i.e., the GC mode). The first model generates homogeneous crystal nuclei into the crystal surface led by the β relaxation. The second model makes assumptions that the tension itself makes free volume, therefore increasing mobility and incentivizes the crystallization process. The last model does not support diffusion-controlled growth (often called diffusionless crystal growth), but the crystal grows due to local molecular motions common for the glassy state [83], it is activated near *T_g_* and continues in the glassy state. Sun et al. applied all three models on seven polymorphs obtained from the liquid ROY which presents the top system for the number of coexisting polymorphs of known structures. Their results revealed that only some polymorphs showed growth. The polymorphs prone to crystal growth, changed crystal morphology with temperature, from faceted single crystals near *T_m_* to fiber-like crystals neat *T_g_*. Sun et al. proved that polymorphs that obey diffusionless CG had rates and Ea similar to polymorphic transformation spotted neat glass transition temperature. Crystal grew faster at rates that are 3–4 orders of magnitude, with Ea which was two times smaller than the Ea of polymorph that did not show any CG [83].

Yang et al. considered crystal growth derived equation (based on Equations (10) and (11)) which allowed them to evaluate the stability of amorphous forms under ambient conditions using data obtained under high temperature and high moisture content. The final form of such modified and modernized Avrami equation (Equation (13)) is presented here. For the complete mathematical derivation of Equation (12) please see the reference [73].

(13) $$\alpha \left(t\right)=1-\frac{1}{1+kt^{n}}$$

where *n* describes the dimensionality of crystal growth: rod structure (*n* = 2, *k* = AβJ0), plate structure (*n* = 3, *k* = 2πβ2hJ0), and sphere structure (*n* = 4, *k* = 1/3πβ3Jo), A is the cross-section surface of the rod, h is the thickness of the plate and β is the crystal growth rate constant. Yang et al. utilized Equation (12) for the efavirenz (EFV)-PVP ASD formulation for prediction of recrystallization kinetics. Kinetics factors such as activation energy obtained under different conditions of temperature and moisture, including PVP content were used for the evaluation of recrystallization kinetics. The value of Ea was determined from the slope of lnk vs. 1/T. PVP affects and inhibit the recrystallization process and the amount of PVP proved to be very significant and critical for amorphous stability. For 75% EFV and 25% PVP no crystalline parts were detected after more than 24 months of storage at 72 °C and 53% RH [73].

Feng et al. used the same approach to investigate the most suitable polymers for fenofibrate (FF) ASD formulation. Recrystallization kinetics of fenofibrate with different molecular weights of HPC (hydroxypropylcellulose) was explored. Each polymer used suppressed the crystallization process, but the polymers with the highest molecular weight were more favorable. The recrystallization rate, k, decreased with an increase in polymer content [84]. In the case of naproxen with water-soluble polymer hydroxypropylmethylcellulose acetate succinate LG (HPMCAS-LG), Yoshihashi et al. calculated the induction period of crystallization to predict the storage period. They showed that if naproxen ASD contained more than 90% of HPMCAS-LG at 333 K or 50% of HPMCAS-LG below storage temperature 301 K, the induction period of crystallization would be more than one year [85]. It should be noted that the induction period of crystallization is reciprocal to the nucleation rate,

(14) $$J\left(t\right)=\frac{1}{V}\frac{dN}{dt}$$

(*N* is the number of nuclei). It is a recommendation that polymers with high *T_g_* are more suitable for ASD due to the reduced molecular mobility, and inhibited nucleation and crystal growth [5]. Using the *T_g_* tool, the stability of ASD can be defined as a resistance of glasses to devitrification upon reheating, especially above or near the glass temperature point [86]. Hancock et al. introduces Tg-50K rules for ASD storing, which means that storage temperature for ASD should be at least 50 K lower than *T_g_* temperature since it was observed that there was no detected molecular mobility in this temperature interval [9]. Nevertheless, Yoshioka et al. reported that the indomethacin drug supported the recrystallization process under *T_g_* and had a sufficient molecular mobility even within storage time [87]. Even though it cannot be taken as a general rule, it gives very valuable information regarding molecular mobility and the physical stability of ASD. Determination of activation energy in the *T_g_* region can be really useful for the evaluation of physical and chemical stability of amorphous form with temperature change. We can address the activation energy as one of the most important quantities for structural relaxations in the glass transition range [88]. The activation energy, along with fragility are valuable characteristics of the glass transition region [89]. The fragility is the dependence of molecules motion with temperature in the glass transition temperature range. This parameter is one of the most important for the assessment of nucleation and crystal growth. Fragility is expressed by the following equation (Equation (15)) [90]:

(15) $$m={\left[\frac{d\log x}{d\left(\frac{T_{g}}{T}\right)}\right]}_{T=T_{g}}$$

where *x* stands for viscosity or structural relaxation time, and *T_g_* is glass transition temperature. Fragility is a measure of how far the physical characteristics of ASD are away from Arrhenius linearity. Hence, Equation (16) can be transformed as follows [90]:

(16) $$x\left(T\right)=x_{0}\exp\left(\frac{\Delta E_{a}\left(T\right)}{RT}\right)$$

High value of *m* > 50 denotes that the rate of molecules motion changes with each 10 K, and small values of *m* < 30 refers to the strong glass former in which rate of molecules motions changes with every 25 K. Strong glass formers are closer to Arrhenius behavior [89]. How far a might a system actually deviate from Arrhenius behavior? Wang el al. explored the maximum fragility index for some nonpolymeric glasses and based on the enthalpy relaxation during cooling and heating around the *T_g_* region, it was calculated to be 175 [91]. Other high fragility values were also reported in the literature (Nb_37_Ni_63_ = 121 [92], Li acetate = 115 [91], and Decalin = 145 [93]). Wang et al. theoretically calculated an even higher fragility index (170 and 192) for non-polymeric glass forming liquids, but these have not been yet experimentally confirmed [94]. For the lower limit, Bohmer et al. reported *m* = 16 based on relaxation time and *m* = 15 and *m* = 17 based on viscosity [95]. Some fragility values of drugs based on literature data are as follows: indomethacin (*m* = 58) [96], felodipine (*m* = 66) [96], celecoxib (*m* = 85) [96], loratadine (*m* = 84) [96], droperidol (*m* = 108) [96], ritonavir (*m* = 127) [96], and carbamazepine (*m* = 35) [97].

Baghel et al. pointed out the significance of fragility and crystallization kinetics in predicting the shelf-life of two amorphous drugs dipyridamole (DPM) and cinnarizine (CNZ) using PVP K30 as carrier matrix at 1:1 drug-polymer ratio. Dipyridamole proved to be more stable due to the faster recrystallization ability of cinnarizine. Crystallization kinetics was investigated in isothermal and non-isothermal conditions and solid-state kinetics was applied. In isothermal crystallization kinetics, the Avrami model was used (JMA, Johnson-Mehl-Avrami), and for DPM ASD the Avrami exponent (Equation (13)) decreased with an increase in temperature. Due to these processes, the following crystallization mechanism was proposed: At T = 351 K, *n* > 2.5 (growth of small particles with increasing nucleation rate), at T = 353–358 K, 1.5 < *n* < 2 (growth of particles with decreasing nucleation rate), and at T = 361 K, *n* < 1.5 (diffusion-controlled growth). In CNZ ASD, the crystallization process develops very fast with lower Ea value (21.23 kJ/mol) compared to the DPM ASD (46.76 kJ/mol). In non-isothermal approach model-free and model-fitting method were used and the former (Kissinger-Akahira-Sunose isoconversional method) gave a better insight into the mechanism of amorphous-crystallization kinetics. The model-fitting method failed to identify the most suitable model since in the case of cinnarizine ASD several models gave a similar correlation coefficient values, while for dipirydamole ASD none of the fitted models could describe the crystallization kinetics properly [98].

It is not uncommon in the literature, that reports on fragility indices of amorphous pharmaceuticals show deviations. For example, for indomethacin, depending on the method used different fragility indices were reported: 76.7 (conventional/modulated temperature differential scanning calorimetry, DSC), 67 (dielectric relaxation spectroscopy, DRS) and 64 (thermally stimulated depolarization currents, TSDC) [99,100]. Recently, Ramos et al. determined the activation energy at *T_g_* and the fragility index of indomethacin using heating rate effect (4–16 °C/min) on the temperature position (*T_m_*) and on the intensity I (*T_m_*) of thermally stimulated depolarization current peak (TS-TSDC) [101]. Values for *E_a_* and *m* were initially calculated based on Equations (15) and (16) (*T_m_* = 315.5 K, *E_a_* = 386 kJ/mol and *m* = 64) and then Equation (17) was used to determine activation energy of a TS peak from the heating rate effect on the temperature *T_m_* (maximum intensity of the peak) and Equation (18) (heating rate effect on the intensity of the maximum of the TS peaks) in order to determine the fragility index of indomethacin.

(17) $$\ln\frac{T_{m}{}^{2}}{r}=\frac{E_{a}}{R}\times \frac{1}{T_{m}}+a$$

(18) $$\ln I\left(T_{m}\right)=-\frac{E_{a}}{RT_{m}}+const$$

*E_a_* was quantified to be 309 kJ/mol and *m* = 51 (Equation (17)) and *E_a_* = 379 kJ/mol and *m* = 63 (Equation (18)). The TS peak obtained by the TSDC technique shows its maximum intensity at a temperature *T_m_* which could be equalized with *T_g_* and as the heating rate increased, *I* and *T_m_* of the TS peaks shifted to higher temperatures. Hancock et al. also developed one pragmatic test for estimating the fragility of amorphous forms of pharmaceutical materials by calculating the *E_a_* for molecules motions on *T_g_* using calorimetric method. Constructing the log of scan DSC rate versus reciprocal *T_g_* value, *E_a_* was calculated from the slope of the best linear fit [89]. A disadvantage of this approach is a necessity for the calibration for each type of material. Similar to this approach, Moynihan et al. also introduced another method (Equation (19)) for *E_a_* determination by DSC using the temperature of onset and end of the glass transition [102].

(19) $$\Delta E_{a}=\frac{CRT_{g}^{onset}T_{g}^{offset}}{\Delta T_{g}}$$

where Δ*T_g_* is the glass transition width, given as Δ*T_g_* = Δ*T_g_^offset^* − Δ*T_g_^onset^* and *C* is a constant. Svoboda et al. researched “How to determine activation energy of glass transition” and presented the advantages and disadvantages of methodologies used for determination of apparent *E_a_* in glass transition. Starting from the Tool-Narayanaswamy-Moynihan Equation which is the most used for evaluation of structural relaxation behavior, he accounted for the effects of thermal history via cooling and heating during cyclic experiments in glass transition range. Cycling experiments were in constant heating rate cycles (Equation (20)) and intrinsic cycles regime (Equation (21)). The first one implied varying cooling rates, while the heating rates remained constant, the other regime, the intrinsic one, implied the opposite.

(20) $$-\frac{\Delta E_{a}}{R}={\left[\frac{d\ln q^{+}|}{d\left(1/T_{p}\right)}\right]}_{\frac{q^{-}}{q^{+}}=const}$$

(21) $$-\frac{\Delta E_{a}}{R}=\left[\frac{d\ln q^{-}|}{d\left(1/T_{f}\right)}\right]$$

In Equation (20), *T_p_* is the temperature corresponding to the maximum of the relaxation peak, *T_f_* (Equation (21)) corresponds to the *T_g_* value obtained on cooling, and *E_a_* is the apparent activation energy of structural relaxation. The best and the most reliable results for *E_a_* were obtained by the intrinsic cycles. It was shown that the errors associated with the determination of apparent activation energy were in between 1–4% (highest errors occurred for lowest *E_a_*). Svoboda et al. presumed that the error origin might be addressed to similar *T_g_* and *T_p_* behavior concerning the heating rate and the intrinsic method which was characterized as a robust one taking into account various data-distortive effects [88].

### 1.4. Stability of Amorphous Solid Dispersion: Hot melt extrusion vs. Spray drying method

Hot melt extrusion (HME) and spray drying (SD) are among the most employable methods for ASD design. Recent advances in HME and SD expedited commercial and industrial application of amorphous solid dispersion concepts for poorly soluble drugs [67,70,103,104]. Examples of commercially available drugs include HME (lumacaftor/HPMCAS/SLS, posaconazole/HPMCAS, griseofluvin/PEG, ritonavir/PVP/PA, lopinavir/PVP/VA), and SD (telaprevir/HPMCAS, etravirine/HPMC, itroconazol/HPMC, tacrolimus/HPMC, rosuvastatin/HPMC) [105,106]. The overviewed and quantified information on molecular mobility, crystallization behavior and component miscibility are of great importance for successful formulation of amorphous solid dispersions by HME and SD. From the crystallization point of view, the HME method results in products with low specific surface area and a high bulk density, while spray drying method gives low bulk density and a high specific surface area [103]. It should be noted that a high surface area is prone to moisture absorption, and therefore can lead to the recrystallization process in ASD. The amorphization during spray drying process is quite stable, as long as the polymers keep the drug away from crystallization and the drying rate is rapid. In the case of hot melt extrusion a high temperature is needed to achieve miscibility between the drug and the polymer, and it is quite challenging to keep HME ASD amorphized when cooled to room temperature. Here, the polymer plays a major role which kinetically inhibits the recrystallization process [103]. A favorable feature for HME is uniform distribution of the drug in polymer which additionally stabilize ASD. Moreover, SD ASD are less homogeneous [107].

Regarding the thermal stability, only drugs that are not heat sensitive can be formulated by HME. On the other hand, the spray drying method is quite suitable for thermo labile drugs and helps in prevention of the thermal decomposition of drugs [14]. Surasarang et al. reported on the albendazole drug formulation via the HME and SD method. The HME method induced almost complete thermal decomposition of albendazole (up to 97,4%), while the spray drying method successfully produced amorphous form of albendazole. Hence, for thermo labile drugs, HME is questionable and challenging method [108]. There are two solutions to deal with this problem and maintain the thermal stability of drugs. The first one is to depress the melting point of a drug and the second is to lower the viscosity of polymer and glass transition temperature, also known as the plasticizing effect [109].

In order to depress the melting point, interactions between the drug and excipients are crucial. Strong drug-polymer interactions, especially ionic interactions and hydrogen bonds, are helpful in improving the drug thermal stability during HME, enhancing the physical stability of ASD during storage. It should be noted that ionic interactions have a typical bond energy around 850–1700 kJ/mol, hydrogen bonds 10–170 kJ/mol, dipole-dipole interactions of 2–8 kJ/mol and a Van der Waals force of ~1 kJ/mol. Liu et al. reported successful preparation of the heat-sensitive drug, carbamazepine by forming cocrystals, via hydrogen bonds between carbamazepine and nicotinamide. The cocrystal formulation had a lower *T_m_* (160 °C) than pure carbamezapin (190 °C) and thermal degradation was avoided [109]. Guo et al. also used drug-polymer interactions to enhance the thermal stability of diflunisal. Combining four polymers as the donors and acceptors of hydrogen bonds, the melting point of heat sensitive diflunisal drug *T_m_* was decreased by almost 55 °C [110]. Kindermann et al. reported a decrement of the melting peak of crystalline naproxen from 154 °C to 120 °C due to ionic interactions between the protonated dimethyl amino groups in Eudragit EPO and the carboxylic acid groups in naproxen [111]. Andrews et al. used interaction between drug and excipients to adjust the *T_g_* point. In physical mixture between bicalutamide and PVP, *T_g_* was decreased with a higher drug loading, indicating that bicalutamide, PVP interactions were stronger then the intermolecular interactions between pure molecules of PVP and bicalutamide due to decreased chain mobility [112]. In contrary, Six et al. reported on HPMC and itraconazole solid dispersion in which the *T_g_* point showed depression due to week interactions between polymer and drug [113]. Carbon dioxide is also known as a plasticizer agent, that can reduce glass transition temperature in many amorphous and semi-crystalline polymers. An antiplasticization effect, in which *T_g_* of the drug-polymer mixture is much higher than *T_g_* of a pure polymer was also reported [114].

Beside formulation, progress in HME methodology can be perceived from technological and processing optimization. Studies that deal with optimization parameters, such as, machine setup, temperature, screw design and screw speed are not rare [115,116,117]. As a successful example of this approach, a case of thermally labile drug glicazide through hot melt extrusion was reported. It is worth mentioning here, in light of the previous section, that thermal stability of glicazide was evaluated based on the Arrhenius equation, which served as a guide for the extrusion optimization process [118].

## 2. Conclusions

Stability of amorphous solid dispersions is one of the most intriguing and investigating topic in the field of the drug development. An overview of thermal stability provided here highlights its intrinsic characteristic connected with drug stability, both chemical and physical. Overall, thermal stability is important for the thermal behavior of drug, thermal decomposition of drug and its products, compatibility/incompatibility between API and excipients, and solubility potential and its shelf-life, which are the most important challenges in the drug development. Furthermore, one should not take for granted that the drug-polymer interaction does not affect the thermal stability of drug. This review aimed to put an applicative feature of solid-state kinetics, which might give quantified predictions of stability of ASD via kinetic and thermodynamic contributions, since activation energy and fragility can be used as successful indicators for poor stability of ASD.

## Figures and Tables

**Table 1:** Set of various solid-state reaction models (differential and integral forms) for thermal transformations in solids [34].

Models	*f*(*α*)	*g*(*α*)
*Nucleation*		
Power law		
P3	2α^1/2^	α^1/2^
P3	3α^2/3^	α^1/3^
P4	4α^3/4^	α^1/4^
*Avrami-Erofe’ev*		
A2	2(1 − α)[−ln(1 − α)]^1/2^	[−ln(1 − α)]^1/2^
A3	3(1 − α)[−ln(1 − α)]^2/3^	[−ln(1 − α)]^1/3^
A4	4(1 − α)[−ln(1 − α)]^3/4^	[−ln(1 − α)]^1/4^
*Geometrical Contraction*		
R2 (contracting area)	2(1 − α)^1/2^	[1 − (1 − α)^1/2^]
R3 (contracting volume)	3(1 − α)^2/3^	[1 − (1 − α)^1/3^]
Reaction−order		
F0 or R1	1	α
F1	(1 − α)	−ln(1 − α)
F2	(1 − α)^2^	(1 − α)^−1^ − 1
*Diffusion*		
D1	1/2α	α^2^
D2	1/[−ln(1 − α)]	(1 − α)ln(1 − α) + α
D3	3(1 − α)^2/3^/2[1 − (1 − α)^1/3^]	[1 − (1 − α)^1/3^]^2^
D4	3/2[(1 − α)^−1/3^ − 1]	(1 − 2α/3) − (1 − α)^2/3^

**Table 2:** Thermal stability of different classes of drugs evaluated by kinetic analysis of solid-state decomposition.

Drug/Class	Arrhenius Parameters	Conclusion	Ref.
Metrodinazol (Anti-micotic drug)	k_A_ = 1.03 × 10^−6^·s^−1^, k_B_ = 16 × 10^−6^·s^−1^, k_C_ = 1.08 × 10^−6^·s^−1^, k_drug_ – 1.03 × 10^−6^·s^−1^	Thermal stability of metrodinazol and its formulation was evaluated based on the rate constants, from Arrhenius classical equation for first−order kinetic indicating the following sequence of thermal stability: Tablet A > Tablet C > Metronidazole drug > Tablet B.	[53]
Losartan potassium (Anti-hypertensive drug)	Ea = 243 kJ/mol, A = 1.65 × 10^17^·s^−1^, *n* = 1	The kinetic studies of the first decomposition step of the two drugs showed a thermal behavior characteristic to first order and indicate that losartan is more thermally stable than valsartan.	[40]
Valsartan (Anti-hypertensive drug)	Ea = 47.31 kJ/mol, A = 2.72 × 10^2^·s^−1^, *n* = 1		
Verapamil hydrochloride (Anti-arrythmic drug)	Ea (drug) = 89.4 kJ/mol, t_10_ = 6.7 yr Ea (form.) = 74.4 kJ/mol, t_10_ = 6.8 years	Verapamil hydrochloride showed thermal stability up to 180 °C, followed by total degradation. Assessing the isothermal degradation kinetics of the drug and formulation, neat drug showed higher thermal stability.	[54]
Acyclovir (Anti-herpetic drug)	Ea = 85.6 kJ/mol, logA = 13.26 s^−1^, k = 5.12 × 10^−20^·s^−1^	Based on the results of degradation kinetics and predicted shelf lives, it could be concluded that acyclovir is an extremely thermally stable drug. However, zidovudine has lower stability and its shelf life is dependent on its storage temperature.	[55]
Zidovudine (Anti-virus drug)	Ea = 117.5 kJ/mol, logA = 11.64 s^−1^, k = 1.10 × 10^−9^·s^−1^		
Cetirizine (Antihistamine drug)	^*^ Ea = 120.8 kJ/mol, logA = 13.4 s^−1^ ^**^ Ea = 122.4 kJ/mol, logA = 13.6 s^−1^ * ASTM, ** Ozawa method	The values of kinetic parameters of simvastatin are about 1.5 times higher than the values for cetirizine. These results show that cetirizine in comparison with simvastatin is a heat sensitive drug, has a shorter shelf-life, and needs more care during storage period.	[56]
Simvastatin (Anticholesteremic drug)	^*^ Ea = 170.9 kJ/mol, logA = 16.0 s^−1^ ^**^ Ea = 171.5 kJ/mol, logA = 16.1 s^−1^ * ASTM, ** Ozawa method		
Naproxen (Anti-infkammatory drug)	^*^ Ea = 81.4 kJ/mol, logA = 7.1 s^−1^ ^**^ Ea = 86.7 kJ/mol, logA = 7.6 s^−1^ * ASTM, ** Ozawa method	The values of kinetic parameters (activation energy and frequency factor) of celecoxib are about 1.5 times higher than those values for naproxen. These results show that naproxen in comparison with celecoxib is a heat-sensitive drug and needs more care during the storage period.	[3]
Celecoxib (Anti-infkammatory drug)	^*^ Ea = 130.9 kJ/mol, logA = 10.5 s^−1^ ^**^ Ea = 134.1 kJ/mol, logA = 10.8 s^−1^ * ASTM, ** Ozawa method		
Efavirenz (Antiretroviral drug)	Ea = 75.230 kJ/mol, A = 3.129 × 10^−16^·s^−1^	Solid decomposition models show similar curves to the nervous impulse. Kinetic study by thermal decomposition of antiretroviral drugs, Efavirenz and Lamivudine can be done by individual adjustment of the solid decomposition models and the use of Kinetic models as activation functions of neurons in the hidden layer.	[52]
Lamivudine (Antiretroviral drug)	Ea = 103.25 kJ/ mol, A = 2.5587 × 10 ^−3^·s^−1^		
Furosemid (Diuretic drug)	Ea = 61.93 kJ/mol	The melting behavior of furosemide and the decomposition products formed during thermal decomposition gave considerable insight into the reasons for the poor aqueous solubility of this drug.	[57]
Gemcitabine	Ea = 123.4 kJ/mol, A = 3.80 × 10^10^ min^−1^ (N_2_ atmosphere) Ea = 126.3 kJ/mol, A = 7.94 × 10^10^ min^−1^ (air)	The thermal decomposition of GTB is a three-stage process. The kinetics shows that the GTB has very good thermal stability. It can be preserved for long-term storage under normal temperature and dry air atmospheres.	[58]
Hydroxytyrosol (Antitumor drug)	E = 128.50 kJ·mol^−1^ ln A = 24.39 min^−1^	Thermal decomposition of HT was completed in the temperature interval between 262.8–409.7 °C. The non-isothermal thermal decomposition mechanism of HT was a 1D diffusion. Mechanism of thermal decomposition was proposed, internal chemical bonds of HT were broken producing small molecules and volatile products.	[7]
Indomethacin Anit-inflammatory drug	Ea = 116.78 kJ/mol MeOH Ea = 69.48 kJ/mol (TBC)	Kinetics of desolvation of IMC in MeOH and TBA, (tertiary butyl alcohol solvate) were studied using isothermal conditions in temperature interval between 60–100 °C. Desolvation followed a nucleation-limited mechanism as described by Avrami-Erofeev. Ea of desolvation whereas was found to be significantly higher for the MeOH, when compared to the TBA solvate. Greater energy has to be supplied to effect the liberation of methanol from the crystal lattice.	[59]
Perindopril (Anti-hypertensive drug)	Ea_neat_ = 59–69 kJ/mol Ea_tablet_ = 170 kJ/mol	Thermal stability and decomposition kinetics of perindopril erbumine as a pure and it tablets were analyzed. The main thermal degradation step of perindopril erbumine is characterized by Ea between 59 and 69 kJ/mol (depending on the method used), while for the tablet, the values were around 170 kJ/mol. The used excipients increased the thermal stability of perindopril erbumine.	[50]
